# Extensive Epiglottitis With Epiglottic Abscess Managed Conservatively: A Case Report

**DOI:** 10.7759/cureus.117757

**Published:** 2026-10-11

**Authors:** Manar Almutiri, Balqees Y Alghanem, Mohamed Zahran

**Affiliations:** 1 General Surgery, Farwaniya Hospital, Al Farwaniyah, KWT; 2 Otolaryngology - Head and Neck Surgery, Kuwait Institute for Medical Specialization, Kuwait City, KWT; 3 Otolaryngology - Head and Neck Surgery, Alexandria University, Alexandria, EGY

**Keywords:** adult epiglottitis, airway management, conservative management, corticosteroids, epiglottic abscess, incision and drainage, intravenous antibiotics, supraglottitis, upper airway obstruction

## Abstract

Epiglottic abscess is a rare but potentially life-threatening complication of adult epiglottitis that can rapidly progress to upper airway obstruction. Although surgical drainage has traditionally been recommended, the optimal management of clinically stable patients remains uncertain. We report a case of a 55-year-old female with no significant medical or surgical history who presented with a three-day history of progressive dysphagia, odynophagia, muffled voice, reduced oral intake, and mild left-sided neck swelling. She was clinically stable, with no stridor, dyspnea, drooling, or hypoxia. Flexible fiberoptic laryngoscopy demonstrated marked supraglottic edema with preserved bilateral vocal cord mobility and an adequate glottic airway. Laboratory investigations showed marked leukocytosis and inflammation (WBC: 20 × 10⁹/L; CRP: 212 mg/L), while contrast-enhanced computed tomography revealed a rim-enhancing epiglottic collection extending to the floor of the mouth, consistent with an epiglottic abscess. The patient was admitted to the intensive care unit and treated conservatively with intravenous ceftriaxone, clindamycin, dexamethasone, and close airway monitoring, with surgical drainage and airway intervention reserved for clinical deterioration. Within 48 hours, she showed marked clinical improvement, normalization of the leukocyte count, a reduction in CRP, and improvement in supraglottic edema on repeat laryngoscopy. Surgical intervention was not required, and she was discharged on oral antibiotics with complete resolution of symptoms. This case highlights that conservative management may be successful in carefully selected adults with epiglottic abscess, particularly when the airway remains stable and there is an early clinical response to treatment. However, given the potential for rapid deterioration, conservative management requires close airway monitoring, serial reassessment, and immediate access to surgical drainage and definitive airway intervention.

## Introduction

Epiglottic abscess is a localized fluid collection involving the vallecula and epiglottis, often associated with diffuse edema of the epiglottis and aryepiglottic folds [1]. It may result from a primary infection, spread from adjacent structures, or infection of a pre-existing mucocele, most commonly on the lingual surface [2]. It is a rare complication of epiglottitis, occurring in about 4% of cases, and is now less frequently seen due to the widespread use of antibiotics and improved diagnostic methods [3]. Despite this, it remains a serious condition with reported mortality rates of up to 30%, mainly due to complications such as airway obstruction, abscess rupture, and hemorrhage [4]. Patients typically present with fever, sore throat, odynophagia, dysphagia, and voice changes, which may progress to dyspnea, stridor, and drooling [3]. Diagnosis is supported by endoscopic findings and confirmed with contrast-enhanced CT, which usually shows a rim-enhancing lesion with central hypoattenuation [4]. Management includes securing the airway, antibiotics, corticosteroids, and drainage when needed, with close follow-up to ensure resolution [2]. Although surgical drainage has traditionally been recommended for epiglottic abscess because of the risk of rapid airway deterioration, selected clinically stable patients may respond to conservative treatment with close monitoring.

Given the potentially life-threatening nature of epiglottic abscess and the lack of consensus regarding optimal management, we report a case of extensive supraglottic infection with epiglottic abscess that was successfully managed conservatively without surgical intervention.

## Case presentation

A 55-year-old female with no known medical or surgical history presented to the emergency department with a three-day history of progressive dysphagia, odynophagia, muffled voice, and reduced oral intake. She also reported mild left-sided neck swelling. There was no history of fever, dyspnea, upper respiratory tract infection symptoms, recent travel, or dental pain.

On initial assessment, the patient was vitally stable and maintaining adequate oxygen saturation on room air. She exhibited no stridor, respiratory distress, or drooling. Oral and oropharyngeal examination showed no trismus and minimal elevation of the floor of the mouth. There was noticeable left parapharyngeal swelling and erythema, with medial displacement of the ipsilateral tonsil and a centrally positioned uvula.

Neck examination demonstrated a firm, tender left submandibular swelling measuring approximately 1 × 1 cm.

Flexible fiberoptic laryngoscopy showed congested nasal mucosa and marked edema of the supraglottic structures. The vocal cords were bilaterally mobile with an adequate glottic airway (Figure 1).

**Figure 1:**
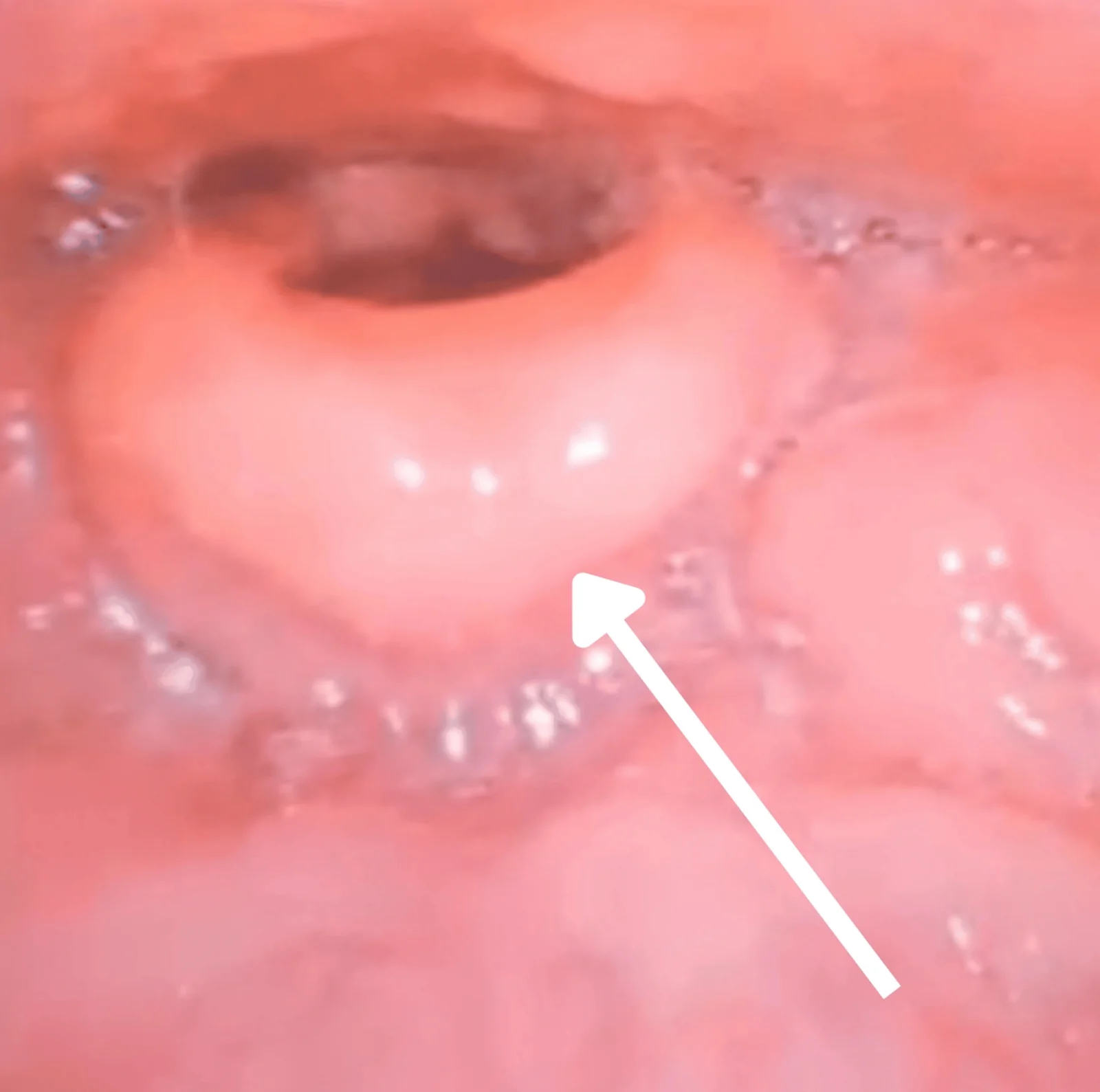
Flexible fiberoptic laryngoscopy demonstrating marked supraglottic and epiglottic edema with significant enlargement of the epiglottis and narrowing of the supraglottic airway.

Laboratory investigations revealed leukocytosis (WBC: 20 ×10⁹/L) with neutrophilia (87%) and markedly elevated inflammatory markers (CRP: 212 mg/L) (Table 1). A contrast-enhanced CT scan of the neck performed at the referring hospital demonstrated a rim-enhancing collection involving the epiglottis with extension to the floor of mouth, consistent with abscess formation (Figure 2).

**Table 1:** Laboratory findings on admission. MCV: mean corpuscular volume; MCH: mean corpuscular hemoglobin; MCHC: mean corpuscular hemoglobin concentration; abs: absolute.

	Value	Reference
White blood cells (×10⁹/L)	20.8	04-10
Red blood cells (×10 ¹²/L)	4.07	3.8-4.8
Hemoglobin (g/dL)	124	120-150
Hematocrit (L/L)	13.4	0.36-0.46
MCV (fL)	90	83-101
MCH (pg)	31	27-32
MCHC (g/dL)	339	315-345
Platelets (×10⁹/L)	237	150-410
Neutrophils (%)	87.5	40-80
Eosinophils (%)	0	1-6
Basophils (%)	0.4	<1-2
Lymphocytes (%)	4.8	20-40
Monocytes (%)	7.2	2-10
Neutrophils (abs) (×10⁹/L)	18.2	2-7
Lymphocytes (abs) (×10⁹/L)	1	1-3
Monocytes (abs) (×10⁹/L)	1.5	0.2-1
Eosinophils (abs) (×10⁹/L)	0	0.02-1
Basophils (abs) (×10⁹/L)	0.08	0.02-0.1
CRP (mg/L)	212.1	0-5

**Figure 2:**
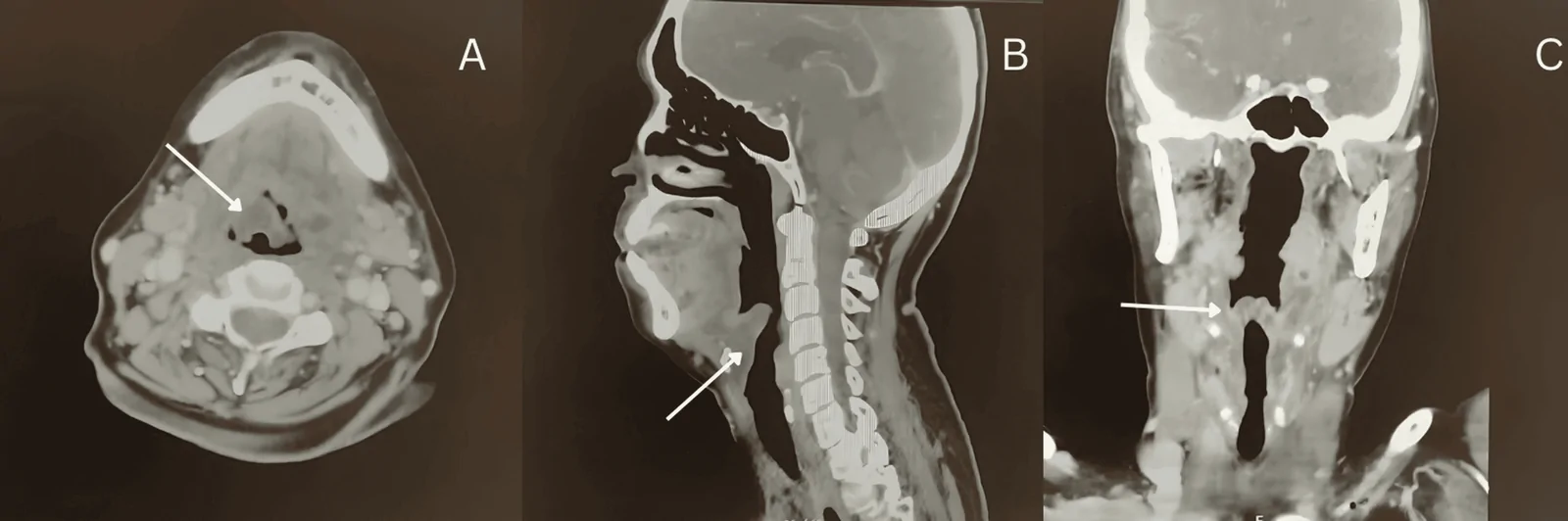
Contrast-enhanced CT of the neck demonstrating marked thickening and edema of the epiglottis with a central rim-enhancing hypodense collection, consistent with an epiglottic abscess. (A) Axial view showing the epiglottic collection and surrounding soft-tissue edema. (B) Sagittal view demonstrating pronounced epiglottic thickening with extension of the inflammatory process inferiorly. (C) Coronal view demonstrating the extent of the supraglottic inflammatory changes.

The patient was admitted to the intensive care unit with a working diagnosis of acute epiglottitis with suspected epiglottic abscess. Given the absence of airway compromise and stable clinical status, she was managed conservatively with close airway monitoring, intravenous ceftriaxone and clindamycin, along with intravenous dexamethasone and omeprazole. A plan was made for possible operative intervention, including laryngoscopy and incision and drainage, with consideration of airway protection via intubation or tracheostomy if required.

Over the subsequent 48 hours, the patient demonstrated significant clinical improvement. Repeat laboratory investigations showed normalization of leukocyte count (WBC: 8.8 ×10⁹/L) and a downward trend in CRP (120 mg/L). Follow-up flexible laryngoscopy revealed improvement in supraglottic edema. Given her favorable clinical course, she did not require surgical intervention and was subsequently stepped down from the intensive care unit. Later, she was discharged on oral antibiotics and remained asymptomatic on follow-up.

## Discussion

Epiglottitis is a potentially life-threatening infection of the supraglottic structures and represents the most common cause of laryngeal abscesses, accounting for approximately 61% of cases in a 34-year review [4]. Although historically more common in children, it remains an important cause of laryngeal abscess formation in adults, with abscess formation reported in 12-19% of adult cases. Several risk factors have been associated with abscess formation, including poorly controlled diabetes mellitus, HIV infection, autoimmune disorders, and immunosuppressive therapy; however, cases without any identifiable predisposing factors have also been described [4]. The main concern in adult epiglottitis is progressive upper airway obstruction, and securing the airway remains the primary priority. Nevertheless, airway intervention is not required in every adult patient. In a systematic review and meta-analysis of 56 studies involving 10,630 adults with epiglottitis, Booth et al. reported an overall airway intervention rate of 15.6%, with tracheal intubation required in 10.2% of patients. The rate of airway intervention also decreased from approximately 20% in earlier decades to around 10% in more recent studies, supporting a more selective approach based on disease severity and the clinical airway status of the patient [5].

Our patient was a 55-year-old female who presented with a three-day history of progressive dysphagia, odynophagia, and muffled voice, together with a marked inflammatory response, with a WBC count of 20 × 10⁹/L and CRP of 212 mg/L. Contrast-enhanced CT demonstrated an epiglottic abscess with extension into the surrounding structures, indicating extensive supraglottic infection. The presence of an abscess is of particular concern because the infection may extend into the pre-epiglottic or paraglottic spaces and may not always be apparent on flexible laryngoscopy. Sideris et al. described two adult cases in which CT identified abscess formation and deep extension despite differing initial airway findings. In one of these patients, the airway was initially unobstructed, but the patient subsequently deteriorated and was found to have a pre-epiglottic abscess requiring surgical drainage and tracheostomy [6]. In contrast, despite the extensive radiological findings in our patient, there were no clinical signs of significant airway compromise, including stridor, dyspnea, drooling, or hypoxia. Flexible laryngoscopy showed marked supraglottic edema, but the vocal cords remained mobile, and the glottic airway was adequate. This stable airway status was an important factor in the decision to continue close observation and conservative treatment. It should also be noted that, although Sideris et al. recommended surgical treatment in the presence of an abscess, their report consisted of only two cases and therefore does not establish abscess formation as an independently validated predictor of airway intervention [6].

The management of epiglottic abscess remains challenging, particularly when deciding between conservative treatment and surgical drainage. Booth et al. reported that the majority of adults with epiglottitis can be managed without airway intervention, but emphasized that disease progression can be unpredictable and that patients managed conservatively should be observed in an appropriate setting with immediate access to airway intervention if needed [5]. There are also reports demonstrating that conservative treatment may fail. Salman et al. described an adult patient who was initially treated with parenteral antibiotics, steroids, humidified air, and close ICU monitoring, but failed to improve. On reassessment, an epiglottic abscess was identified with approximately 50% reduction in the glottic opening, prompting intubation and surgical incision and drainage, after which the patient improved rapidly [7]. On the other hand, Eta recently reported successful conservative treatment of a 30-mm epiglottic abscess with extension toward the tonsillar region using intravenous antibiotics, corticosteroids, supportive care, and close ICU airway monitoring, without surgical drainage [8]. These contrasting outcomes suggest that conservative treatment can be successful in selected patients, but requires careful patient selection, close monitoring, and repeated assessment to identify early treatment failure or deterioration.

What makes our case notable is the successful conservative management of an extensive epiglottic abscess despite the significant inflammatory response and radiological extension. The decision to continue medical treatment was based not simply on the absence of stridor, but on the overall clinical picture, including a stable airway on flexible laryngoscopy, absence of respiratory distress or hypoxia, and subsequent objective improvement. Within 48 hours, the patient showed marked clinical improvement, normalization of the WBC count, a reduction in CRP, and improvement in supraglottic edema on repeat laryngoscopy. She therefore avoided surgical drainage and airway intervention and was ultimately discharged without complications. Taken together with previous reports of both successful and failed conservative management, our case supports the consideration of conservative treatment in carefully selected adults with a stable airway and early clinical improvement, provided that close airway monitoring and immediate access to surgical intervention are available.

## Conclusions

Epiglottic abscess in adults is a rare but potentially serious condition with the risk of sudden airway compromise. Although surgical drainage remains an important option, this case shows that conservative treatment can be successful in carefully selected patients with a stable airway and early clinical improvement. In our patient, close ICU monitoring, intravenous antibiotics and corticosteroids, together with repeated clinical and endoscopic assessment, allowed recovery without the need for drainage or airway intervention. However, conservative management should only be considered when close airway observation is possible and surgical intervention can be performed promptly if the patient deteriorates or fails to improve.
